# Effects of three different types of exercise on blood leukocyte count during and following exercise

**DOI:** 10.1590/S1516-31802003000100003

**Published:** 2003-01-02

**Authors:** Valéria Maria Natale, Ingrid Karen Brenner, Andrei Ion Moldoveanu, Paris Vasiliou, Pang Shek, Roy Jesse Shephard

**Keywords:** Endurance, Exercise, Resistance, Immune cells, Inflammation, Exercício, Resistência, Cardiovascular, Células, Sistema, Imune, Inflamação

## Abstract

**CONTEXT:**

High-intensity exercise causes tissue damage, production of stress hormones, and alterations in the function and quantity of various immune cells. Many clinical-physical stressors such as surgery, trauma, burns and sepsis induce a pattern of hormonal and immunological response similar to that of exercise. It has thus been suggested that heavy exercise might be used to cause graded and well-defined amounts of muscle trauma, thereby serving as an experimental model for inflammation and sepsis.

**OBJECTIVE:**

In order to explore whether some form of strenuous exercise might provide an useful model for the inflammatory process, we studied the effects of three different exercise protocols on blood leukocyte count during and following exercise.

**DESIGN:**

Four different experimental conditions, using a randomized-block design.

**SETTING:**

Defence and Civil Institute of Environmental Medicine, North York, Ontario, Canada.

**PARTICIPANTS:**

Eight healthy and moderately fit males.

**PROCEDURES:**

Participants were each assigned to four experimental conditions. Subjects performed 5 minutes of cycle-ergometry exercise at 90%, 2 hours of cycle-ergometry exercise at 60%, a standard circuit of resistance exercises with 3 sets of 10 repetitions at 60 to 70% of one-repetition maximum (1-RM) force at each of 5 different stations; or they remained seated for 5 hours.

**DIAGNOSTIC TEST USED:**

Flow cytometric analysis.

**MAIN MEASUREMENTS:**

Blood samples were analyzed for total leukocyte counts, total T cells, T helper/inducer cells, T suppressor/cytotoxic cells, B cells, cytolytic T cells, and natural killer cells.

**RESULTS:**

The peak aerobic and prolonged submaximal exercise induced similar alterations in cell counts. These changes were generally larger than those produced by the resistance exercise, although both resistance and peak aerobic exercise resulted in a significantly longer-lasting decrease in the CD4 / CD8 ratio than the submaximal exercise bout did.

**CONCLUSION:**

The data suggest that, of the three exercise patterns tested, prolonged aerobic exercise induced the largest and most readily measured patterns of immune response. Nevertheless, the changes provided only a partial model for the clinical inflammatory process.

## RESUMO

**CONTEXTO:**

Exercícios físicos muito intensos causam dano tecidual, produção de hormônios do estresse e alterações do número e função de diversas células do sistema imune no sangue circulante. Muitos estímulos indutores de estresse, clínicos e físicos, tais como cirurgia, trauma, queimaduras e septicemia, induzem um padrão de resposta hormonal e imunológica semelhante àquela induzida pelo exercício. Existem evidências de que o exercício extenuante poderia ser um método a ser utilizado para causar quantidades variadas e bem definidas de trauma muscular, servindo, assim, como um modelo experimental para o estudo da resposta inflamatória e da septicemia.

**OBJETIVO:**

Estudamos os efeitos de três diferentes protocolos de exercício sobre o número dos leucócitos sangüíneos com o objetivo de investigar se alguma forma de exercício extenuante poderia fornecer um modelo útil da resposta inflamatória.

**TIPO DE ESTUDO:**

Estudo randomizado, utilizando quatro diferentes condições experimentais.

**PARTICIPANTES:**

Foram estudados oito indivíduos do sexo masculino, saudáveis e com preparo físico moderado (média de idade: 24,9 ± 2,3 anos).

**PROCEDIMENTOS:**

Foram atribuídas quatro condições experimentais, uma por vez, usando um modelo de distribuição randomizada. Os indivíduos realizaram cinco minutos de exercício em bicicleta ergométrica a uma intensidade de 90% do VO_2max_, duas horas de exercício em bicicleta ergométrica a uma intensidade de 60% do VO_2max_, um circuito padrão de exercícios de resistência (3 sets de 10 repetições numa intensidade de 60 a 70% de uma repetição máxima em cinco postos diferentes) ou permaneceram sentados durante cinco horas.

**TESTES LABORATORIAIS UTILIZADOS:**

Análise por citometria de fluxo.

**VARIÁVEIS ESTUDADAS:**

As amostras de sangue foram analisadas para a contagem dos leucócitos totais, das células T totais (CD3^+^), dos linfócitos T auxiliares (CD3^+^CD4^+^), dos linfócitos T citotóxicos/supressores (CD3^+^CD8^bright+^), dos linfócitos B (CD19^+^), dos linfócitos T citotóxicos (CD3^+^CD16^+^/56^+^) e das células *natural killer* (CD3^–^CD16^+^/56^+^).

**RESULTADOS:**

Os exercícios aeróbicos, tanto o de alta intensidade quanto o prolongado submáximo, induziram alterações similares na contagem de células, e essas alterações foram maiores do que as induzidas pelos exercícios de resistência muscular, embora tanto o exercício aeróbico intenso quanto o de resistência muscular tenham resultado num decréscimo significantemente mais prolongado da relação CD4^+^/CD8^+^ do que o exercício aeróbico submáximo.

**CONCLUSÃO:**

Esses dados, em conjunto com nosso estudo paralelo sobre as alterações humorais observadas em indivíduos que realizaram os mesmos exercícios, sugerem que, dentre os três modelos de exercício estudados, o exercício aeróbico prolongado foi o que induziu as maiores e mais evidentes alterações mensuráveis da resposta imune, embora essas modificações somente forneçam um modelo parcial de um processo inflamatório clínico.

## INTRODUCTION

The interrelationships between exercise and immune function have been widely studied since the first publications on exercise-induced leukocytosis.^1,2^ An acute bout of exercise places a wide spectrum of demands on the body, depending on the form, intensity and duration of the required effort, together with physiological and psychological constraints peculiar to the host. High-intensity exercise causes tissue damage, production of stress hormones, and alterations in the circulating quantity and function of various immune cells. Many clinical-physical stressors such as surgery, trauma, burns and sepsis induce a pattern of hormonal and immuno-logical response similar to that of exercise. Specific changes that have been observed, both following strenuous exercise and in infectious disease states, include: the acute phase response, leukocyte mobilization and activation, release of inflammatory mediators (cytokines), tissue damage and cell infiltration, the production of free radicals and activation of the complement, coagulation and fibrinolytic pathways.^2,3^

It has thus been suggested that heavy exercise might be used to cause graded and well-defined amounts of muscle trauma, thereby serving as an experimental model for inflammation and sepsis.^4^ Obviously, the responses to ethically acceptable doses of exercise are much smaller than those seen in sepsis. Therefore, in order to obtain readily measured changes, it is important to choose a pattern of activity that maximizes disturbances in immune function. A parallel investigation has reported changes in cytokines and natural killer cell activity.^5^

In the present study, we compared changes in circulating white cell count and subsets, in the same group of subjects during and after performance of several different types of strenuous exercise (peak aerobic exercise, prolonged endurance exercise, and a standard circuit of resistance exercises). Since the early phases of the immune response to exercise have only had limited examination until now, we also considered that it would be useful to compare the effects of moderately intense versus high-intensity exercise on circulating leukocyte count during the first five minutes of exercise.

## METHODS

***Subjects and experimental design***. Eight healthy male, non-smoking, and relatively sedentary volunteers were recruited under conditions approved by the Human Experimentation Committees of the University of Toronto and the Defence and Civil Institute of Environmental Medicine (DCIEM). They were informed of the purpose, nature and possible side effects involved in the study, and gave their written informed consent.

Each subject visited the laboratory on a total of 13 occasions over the course of 8-9 weeks. Participants were asked to maintain their normal (sedentary) lifestyle throughout this period. The initial two-hour session included a medical examination (which excluded those with recent infection and allergic conditions) and tests of maximal aerobic power (VO_2max_), and one-repetition maximal (1-RM) voluntary force. The latter was measured at five different stations of a resistance exercise circuit: biceps curl, knee station, hamstring curl, bench press and leg press. Physical measurements (height, body mass and skinfolds) were also completed at this visit. The VO_2max_ was determined using a standard incremental cycle ergometer protocol.^6^ Expired gas was collected and analyzed using a SensorMedics Metabolic Measurement Cart (System MTS 4400, Alpha Technologies, Anaheim, California, USA).

Volunteers were assigned to a sequence of four different experimental conditions, using a randomized-block design. Individual experiments began on the same day of the week and at the same time of day, to avoid circadian and circaseptan effects. In one experiment, subjects performed 5 min of self-paced peak aerobic cycle ergometry at an intensity equivalent to 90-97% of the individual's directly measured VO_2max_ (subsequently described as peak aerobic effort, PA). In a second experiment, two hours of cycle-ergometry exercise (Long) were completed at 60 to 65% of the individual's personal VO_2max_. A third experimental session required completion of a standard circuit of resistance exercises (RE) in which three sets of 10 repetitions at 60 to 70% of 1-RM force were completed at each of the five stations noted above. Subjects remained seated for a 3-hour recovery period following each type of exercise. On the control day (Sit), the subjects remained seated at rest for 5 hours.

The subjects had a mean age of 24.9 years (± 2.3), a height of 1.74 m (± 0.24), a body fat content as estimated by the Durnin and Womersley equations^7^ of 16.6% (± 2.2), and a peak aerobic power of 43.0 ml kg^-1^min^-1^ (± 3.1).

The heart rate was monitored continuously throughout each of the four sessions, using a Sport-Tester^®^. The oxygen consumption was also measured intermittently during the two types of aerobic exercise, using the SensorMedics Metabolic Cart. Volunteers were encouraged to drink water during and following all experimental conditions.

***Blood sampling***. Participants arrived at the laboratory after a 12-hour fast, having refrained from vigorous exercise for at least 24 hours. On arrival, they were given a simple standardized breakfast, 1.1 MJ (250 kcal) of a commercial liquid meal supplement (16-oz Ensure Plus, Abbott Laboratories, Saint Laurent, Quebec, Canada). In three of the four conditions (peak aerobic exercise, prolonged exercise and resting control), blood samples were collected from an indwelling heparin-locked latex venous catheter (Deseret Medical, Sandy, Utah, USA). The catheter was inserted into the median antecubital vein 30 minutes prior to collection of the first blood sample. Additional blood samples were collected 5 minutes, 1, 2, 3, 4 and 5 hours later during each of the three sessions. During the resistance exercise routine (RE), samples were collected by venipuncture at the corresponding times. At the 24-hour and 72-hour visits, subjects were questioned about any muscle soreness that had developed, and additional blood samples were collected by venipuncture.

Complete blood cell counts, differential leukocyte counts, and hemoglobin and hematocrit determinations were performed on K3EDTA-treated blood, using an automated Coulter JT hematology analyzer (Coulter Electronics, Hialeah, Florida, USA). All parameters were adjusted for blood volume changes, using the Dill and Costill method.^8^

***Immunophenotyping***. A dual combination of monoclonal antibodies (mAb) conjugated to fluorescein isothiocyanate (FITC) or phycoerythrin (PE) (Becton-Dickinson, Mississauga, Ontario, Canada) was used to enumerate lymphocyte subsets. Stained-cell suspensions were analyzed using a FACScan^®^ flow cytometer (Becton-Dickinson Immunosystems, Mountain View, California, USA). Using FACScomp^®^ software (Becton-Dickinson Immunocytometry Systems), the cytometer was calibrated with a mixture of monosized FITC and PE-conjugated and unconjugated latex particles (4.8 mm Calibrite^®^ beads, Becton-Dickinson). An isotype negative control optimized the settings of the fluorescence detectors for each subject. The fluorescence compensation was adjusted using an anti-CD4mAB (FITC)/anti-CD8 (PE) dual-stained sample. Gating for the lymphocyte populations and boundaries for fluorescence intensity were determined using unstained control samples. The usual quantity of cells scanned was 10,000 cells per sample. Absolute cell numbers were calculated from the total lymphocyte count, as quantified by the Coulter counter, using the equation: total number of "cell type" per liter of blood = [(% "cell type"/100) x (total number of lymphocytes/l blood)].

***Statistical Analyses***. Results are expressed throughout as mean ± standard error (SE). As in other small-sample studies on relatively homogenous populations, a normal distribution of data was assumed. Changes in leukocyte and lymphocyte subsets were analyzed by a repeated-measures analysis of variance involving four conditions and five measurement times (SuperAnova, Abacus Concepts, Berkeley, California, USA). Post-hoc contrasts explored significant main effects and interactions. For all analyses, a probability (p) value of < 0.05 was set as the level of statistical significance.

## RESULTS

***Subject characteristics***. Subjects had a mean age of 24.9 years (± 2.3), a height of 1.74 m (± 0.24), a body fat content as estimated by the Durnin and Womersley equations^7^ of 16.6% (± 2.2), and a peak aerobic power of 43.0 ml kg^-1^min^-1^ (± 3.1).

***Heart rate response.*** All three types of exercise induced a substantial increase in heart rate. Immediately after exercise, the response was greatest for peak aerobic exercise (180 ± 6 beats/min), somewhat less for prolonged exercise (149 ± 7 beats/min), and even less for the resistance exercises (123 ± 11 beats/min).

***Leukocyte Counts***. All three types of exercise provoked leukocytosis that persisted for 3 hours after exercise. The magnitude of this response was graded such that Long [post-exercise: (13.20 ± 1.71) x 10^9^ cells/liter; recovery 3 h: (11.87 ± 1.43) x 10^9^ cells/liter] was greater than PA [post-exercise: (9.63 ± 0.96) x 10^9^ cells/liter; recovery 3 h: (7.80 ± 1.07) x 10^9^ cells/liter], which in turn was greater than RE [post-exercise: (7.71 ± 0.82) x 10^9^ cells/liter; recovery 3 h: (8.11 ± 0.78) x 10^9^ cells/liter], with p-values varying from < 0.01 to < 0.0001. The values differed significantly (p-value varying from < 0.02 to < 0.0001) from the seated control, both immediately and 3 hours after exercise. For seated-control volunteers, the total leukocytes number was measured during the five hours of rest as follows: time zero: (5.49 ± 0.19) x 10^9^ cells/liter; 5 minutes: (5.44 ± 0.13) x 10^9^ cells/liter; 1 hour: (5.50 ± 0.14) x 10^9^ cells/liter; 2 hours: (5.80 ± 0.09) x 10^9^ cells/liter; 3 hours :(6.17 ± 0.22) x 10^9^ cells/liter; 4 hours: (6.27 ± 0.28) x 10^9^ cells/liter; and 5 hours: (6.60 ± 0.27) x 10^9^ cells/liter. After 24 hours of recovery, there were no residual differences among conditions.

As expected, the leukocytosis was due mainly to an increase in the quantity of circulating neutrophils and monocytes, although there was also a small increase in lymphocyte count. The circulating monocyte count fell below the seated control (0.45 ± 0.08) x 10^9^ cells/liter) after 24 hours, following both peak aerobic exercise (0.35 ± 0.05) x 10^9^ cells/liter) and resistance exercises (0.34 ± 0.05) x 10^9^ cells/liter).

**Figure 1:**
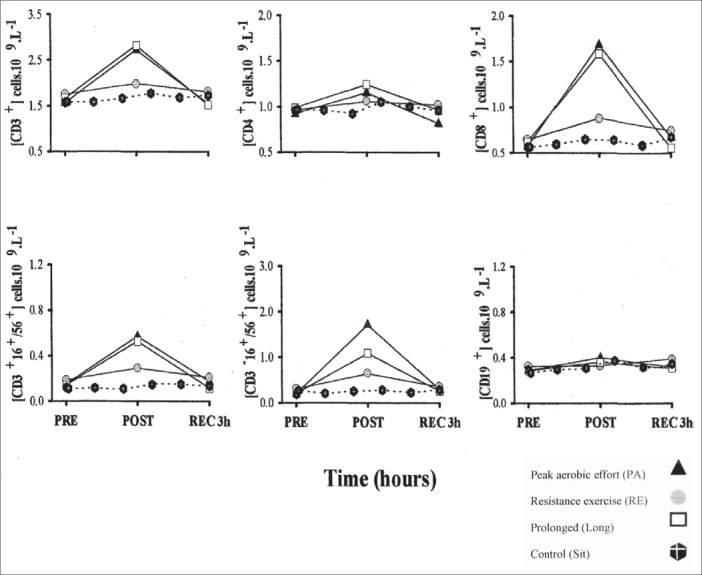
The response of CD3^+^, CD4^+^, CD8^+^, CD19^+^, CD3^+^16^+^56^+^ and natural killer cells to three types of exercise compared with seated rest for the same period of the day. PRE = before exercise, POST = after exercise, and REC = recovery time.

***Lymphocyte Subsets.*** All three types of exercise induced a significant (p varying from 0.02 to 0.0001) increase in total T lymphocyte (CD3^+^) cell counts immediately after exercise (Figure 1). There were similar changes for peak aerobic [(2.73 ± 0.24) x 10^9^ cells/liter] and prolonged exercise [(2.83 ± 0.24) x 10^9^ cells/liter], and a much smaller response to resistance exercise [(1.99 ± 0.13) x 10^9^ cells/liter]. However, by 3 hours after exercise, the cell counts for prolonged exercise had returned to baseline, and the data for peak aerobic exercise were then some 35% below baseline (Figure 1).

The circulating CD4^+^ (T helper) cell counts showed a similar and significant increase with peak aerobic [(1.15 ± 0.13) x 10^9^ cells/liter] and prolonged exercise [(1.24 ± 0.10) x 10^9^ cells/liter], whereas the resistance exercises [(1.06 ± 0.08) x 10^9^ cells/liter] did not augment the CD4^+^ cell count significantly (Figure 1). Counts had returned to baseline by 3 hours after the prolonged exercise, but again, counts were subnormal 3 hours after the peak aerobic exercise.

All three types of exercise induced a significant increase in CD8^+^ (cytotoxic/suppressor) cell count (Figure 1) immediately after exercise, but baseline values were restored by 3 hours after exercise.

The CD4^+^/CD8^+^ ratio was decreased significantly immediately following all three types of exercise, although the change was smaller for resistance exercise than for the other two types of activity. Values for peak aerobic exercise and resistance exercise tended to remain below baseline for up to 24 hours after exercise, but values had increased significantly above baseline by 3 hours after prolonged exercise.

All three types of exercise induced a significant increase in circulating CD3^–^ CD16^+^ CD56^+^ (natural killer) cell counts immediately after exercise (Figure 1). The rise was greatest for peak aerobic exercise [(1.72 ± 0.24) x 10^9^ cells/liter], somewhat less for prolonged exercise [(1.09 ± 0.14) x 10^9^ cells/liter] and much less for resistance exercise [(0.64 ± 0.12) x 10^9^ cells/liter]. However, all values had returned to baseline by 3 hours after exercise.

Peak aerobic [(0.57 ± 0.13) x 10^9^ cells/liter] and prolonged [(0.53 ± 0.14) x 10^9^ cells/liter] exercise promoted similar increases in circulating CD3^+^CD16^+^CD56^+^ cell counts (Figure 1) immediately following activity. The changes were significantly greater than those promoted by resistance exercise [(0.29 ± 0.07) x 10^9^ cells/liter]. However, the decreases in count 3 hours after exercise were greater for peak aerobic [(0.18 ± 0.05) x 10^9^ cells/liter] and prolonged [(0.12 ± 0.03) x 10^9^ cells/liter] exercise than for resistance exercise [(0.21 ± 0.06) x 10^9^ cells/liter].

Exercise induced few changes in CD19^+^ (B) cell counts (Figure 1). Only peak aerobic exercise increased counts immediately after exercise, although resistance exercise also induced a significant increase 3 hours after exercise.

The results for the lymphocyte subtypes 24 hours and 72 hours after exercise are not illustrated, because by that stage none of the data differed from seated control values.

*Data obtained after 5 minutes of exercise*. The differences in leukocyte responses to five minutes of moderate and peak aerobic exercise are summarized in Table 1. Over this period, the peak aerobic exercise induced significantly larger increases in total leukocyte, lymphocyte, CD3^+^, CD8^+^. CD3^–^CD16^+^/56^+^ and CD3^+^ CD16^+^/56^+^ counts than the less vigorous exercise.

**Table 1:** Comparison of circulating leukocyte and lymphocyte subset counts, 5 minutes into peak or prolonged aerobic exercise. Values are means ± standard error (SE)

CIRCULATING WHITE BLOOD CELL COUNT (cells/mm^3^)	PEAK AEROBIC EXERCISE (90-97% VO2_max_)	PROLONGED AEROBIC EXERCISE (60% VO2_max_)
Total leukocytes	9.63 ± 0.96*	8.06 ± 0.51
Lymphocytes	4.86 ± 0.41*	3.65 ± 0.27
Monocytes	0.63 ± 0.10	0.60 ± 0.09
Neutrophils	4.13 ± 0.55	3.99 ± 0.44
CD3^+^	2.73 ± 0.24*	2.31 ± 0.16
CD3^+^CD4^+^	1.15 ± 0.13	1.17 ± 0.09
CD3^+^CD8^+^	1.69 ± 0.25*	1.15 ± 0.15
CD3^+^CD16^+^56^+^	0.57 ± 0.13*	0.36 ± 0.09
CD3¯CD16^+^56^+^	1.71 ± 0.24*	0.86 ± 0.15
CD19^+^	0.40 ± 0.05	0.41 ± 0.07

* *indicates difference between the two types of exercise (p < 0.05).*

## DISCUSSION

In order to explore which type of strenuous exercise provides the most useful model of the inflammatory process, we compared the effects of three different types of exercise on blood leukocyte numbers and body fluid responses both during and following exercise. As discussed previously,^5^ a study of natural killer cell activity and pro-inflammatory cytokine secretion (interleukin-6 and tumor necrosis factor, TNFa) produced during the same three types of exercise indicated that the greatest response was for prolonged aerobic activity. In contrast, delayed muscle soreness and the release of creatine kinase three days after exercise were greatest with resistance exercise.

The changes in circulating cell counts, now to be discussed, also indicated that the greatest response was during and following prolonged aerobic exercise.

***Early response to exercise***. There have been few comparisons made of the responses to differing intensities of exercise during the first five minutes of effort. The present observations indicate that, in relation to moderate exercise (60% of VO_2max_), the peak aerobic exercise (90-97% of VO_2max_) induced a significantly greater increase in circulating counts for total leukocytes, lymphocytes, CD3^+^ lymphocytes, CD8^+^ cytotoxic-suppressor lymphocytes, natural killer cells and CD3^+^CD16^+^/56^+^ cells. Presumably, the more intense exercise induced a greater early rise of catecholamine concentrations, and thus a greater mobilization of the various cell populations. The peak aerobic exercise also induced a significantly more prolonged decrease in the CD4^+^/ CD8^+^ ratio following the bout of activity.

***Cellular response immediately after exercise***. Concurring with several other authors,^9-13^ we observed a significant increase in total leukocyte, neutrophil, monocyte and lymphocyte counts immediately following all three types of exercise. Prolonged exercise induced the greatest increase in total circulating leukocyte, neutrophil and monocyte counts, but peak aerobic exercise induced a similar increase in lymphocyte counts; lesser responses in all cell subsets were provoked by resistance exercise.

During exercise, natural killer, T and B cells are all recruited into the circulating blood stream.^2^ However, the natural killer cell count increases by more than the T cell count, so that the CD3^+^ T cell percentage declines during exercise. The number of CD8^+^ cells also increases by more than the CD4^+^ cells, resulting in a decreased CD4^+^/ CD8^+^ ratio.^14^ In the present study, all three types of exercise induced a significant increase in circulating T lymphocyte (CD3^+^, CD4^+^ and CD8^+^) cell counts immediately after exercise, with peak aerobic and prolonged exercise resulting in similar changes, and the resistance exercises inducing a much smaller response. All three types of exercise caused a significant decrease in the CD4^+^/ CD8^+^ ratio immediately after exercise, but differences in this ratio between the three protocols clearly reflect differing cell responses to the three types of activity.

In agreement with other investigators, the three types of exercise caused distinct yet significantly different increases in natural killer cell count immediately after exercise. Both Kendall et al.^9^ and Nieman et al.^12^ observed an augmentation of the exercise-induced increase in natural killer cell count as the intensity of activity was increased. Presumably, catecholamine secretion and consequently cell demargination increase with the intensity of effort, and there may also be an intensity-related gradation of effects from increased intravascular shear stress. Gabriel et al.^15^ ob-served that the rise in natural killer cell count was correlated with the increase in heart rate; our data confirm this finding (results not shown), which is compatible with either a catecholamine or a shear-stress mediated response. Nieman et al.^16^ studied reactions to 30 seconds of all-out exercise. They found a 176% increase in natural killer cell count immediately after exercise; perhaps the much larger response we observed was due to the fact that our near-maximal aerobic effort continued for 5 minutes. Nieman et al.^16^ demonstrated a natural killer cell count that was 50% below resting values one hour after the same stimulus. We were interested primarily in the longer-term inflammatory response, and we may have missed a change of this type, since our first post-exercise blood sample was collected 3 hours after exercise.

We found only small changes in circulating CD19^+^ B lymphocyte cell counts, in agreement with others who have studied the response to heavy^15-17^ and moderate^18,19^ endurance exercise and resistance exercise.^13,20^

***Evidence for subsequent immunosuppression***. Circulating total leukocyte, neutrophil and monocyte counts remained high for 3 hours after exercise. Circulating lymphocyte counts had returned to baseline by 3 hours after exercise, although we had expected them to fall below baseline, especially following peak aerobic and prolonged exercise. By three hours after exercise, T cell counts had also returned to baseline in the case of prolonged and resistance exercises, but numbers were decreased a little below baseline with the peak aerobic exercise. CD4^+^ cell counts had returned to baseline by 3 hours after prolonged exercise, but with peak aerobic exercise, cell counts fell below baseline at this stage of recovery. CD8^+^ counts had all returned to baseline by 3 hours after exercise. The CD4^+^/CD8^+^ ratio was thus higher than seated control values 3 hours after exercise. Ratios obtained after resistance exercise and peak aerobic exercise remained below the seated control values throughout the 24 hours of recovery (p < 0.001 and p < 0.01 respectively). The resistance exercises caused a smaller decrease in this ratio than the other two types of exercise did, although ratios still remained significantly below baseline 24 hours after exercise.

The extent and duration of any immunosuppression probably depends on both the intensity and duration of the exercise that has been undertaken.^2,21,22^ Nieman et al.^23^ found that 45 minutes of exercise, whether at 50% or 80% of the individual's aerobic power, led to significant lymphocytopenia. This response was somewhat more marked after high-intensity exercise (80% of aerobic power) than with moderate intensity activity (50% of aerobic activity), and the changes persisted for 1 to 3.5 hours after exercise. Our results apparently differ from those of Nieman et al.,^23^ but this could be explained by our choice of a longer period of prolonged exercise (2 hours), the large difference in fitness of the volunteers (aerobic power of 43 versus 66 ml kg^-1^min^-1^), or the timing of the first recovery blood sample (three versus one hour after exercise).

Our results also differed from Nieman et al.^20^ with respect to resistance exercise. Nieman et al.^20^ found that leg squat exercise induced strong leukocytosis, lymphocytosis and lymphocytopenia, similar in magnitude to the changes reported after endurance exercise. However, the subjects in the Nieman study performed resistance exercise on a single muscle group "until muscle failure", whereas in the present study the intensity of exercise was more moderate, and it was distributed over five exercise stations. Moreover, Nieman did not use the same subjects for endurance and resistance exercise, making it difficult to compare responses to the two stimuli in his study. Tvede et al.^13^ compared subjects who performed first endurance and then resistance exercise, but unfortunately this study neglected to balance the order of the two types of exercise. Like us, Tvede et al.^13^ found that endurance exercise induced marked leukocytosis, neutrophilia, lymphocytosis and lymphocytopenia, whereas resistance exercise provoked a significant but smaller response from the same cells.

Our subjects also contributed to the parallel study of Brenner et al.,^5^ in which blood samples obtained during the same series of exercises were analyzed for creatine kinase (CK), natural killer cell counts (CD3^-^/CD16^+^56^+^), cytolytic activity and plasma levels of cytokines (interleukin-6, IL-6, TNF-a, and IL-10). Their results showed that the prolonged exercise induced a significant increase in IL-6 and TNFa plasma levels, the mobilization of cytotoxic cell populations, and increased natural killer cell cytotoxic activity, all suggesting that prolonged exercise was effective in activating several components of the inflammatory response. These data, taken in conjunction with our present results, suggest that among the three different exercise protocols that we examined, prolonged exercise generated the largest inflammatory response. Nevertheless, the changes induced by all three types of exercise peaked long before changes in creatine kinase and muscle soreness, and neither the cell nor the body fluid changes matched the dominant symptomatic and creatine kinase response to resistance exercise.

## CONCLUSIONS

Of the three patterns of exercise studied, prolonged aerobic activity (two hours at 60% of VO_2max_) generated the largest and thus the most readily measured changes in immune response. In contrast, resistance exercise was associated with a greater release of creatine kinase and late-onset muscle soreness. It appears that any ethically acceptable form of vigorous exercise provides only a partial model of a clinical inflammatory response. Future research in this area should seek to relate changes in circulating leukocyte counts and cytokines to hormonal, symptomatic and enzymatic changes.
